# Consistently high accuracy of digital 2D templating in total knee arthroplasty across different levels of surgical training

**DOI:** 10.1186/s42836-025-00356-5

**Published:** 2026-01-06

**Authors:** Moses K. D. El Kayali, Fahad Imtiaz, Luis V. Bürck, Sebastian Braun, Clemens Gwinner, Lorenz Pichler, Rosa Berndt

**Affiliations:** 1 Center for Musculoskeletal Surgery, Charité – Universitätsmedizin Berlin, Berlin, 10117 Germany; 2 Institute for Clinical and Experimental Surgery, Saarland University, Homburg/Saar, 66421 Germany; 3 Department of Orthopedics and Trauma-Surgery, Berlin Jewish Hospital, Berlin, 13347 Germany; 4 Department of Orthopedics and Trauma-Surgery, Medical University of Vienna, Vienna, 1090 Austria

**Keywords:** Total knee arthroplasty, Digital templating, Preoperative planning, Osteoarthritis

## Abstract

**Purpose:**

To evaluate the accuracy of two-dimensional (2D) digital templating in primary total knee arthroplasty (TKA) and assess whether surgical training level affects templating accuracy.

**Methods:**

A total of 424 patients who underwent primary TKA with preoperative 2D digital templating using the Attune system were retrospectively analyzed. Templating was performed in TraumaCad (Brainlab AG) by junior residents (< 3 years of training), senior residents (≥ 3 years), or board-certified orthopaedic surgeons. Planned and implanted component sizes were compared, and accuracy was assessed as exact matches and deviations of ± 1, ± 2, and ± 3 sizes. Pearson correlation analysis was used to assess the association between planned and implanted sizes. One-way ANOVA was used to compare mean absolute deviation across training levels. Additionally, the proportion of cases with a deviation greater than ± 1 size was calculated for both components across experience levels and compared using chi-square tests.

**Results:**

A total of 424 patients (61% female) were included. The median planned component sizes were 6 (IQR, 5–7) for the femoral and 6 (IQR, 5–7) for the tibial component; the median implanted sizes were 6 (IQR, 5–7) and 6 (IQR, 4–7), respectively. Planned and implanted sizes were very strongly correlated for both femoral (*r* = 0.864; *P* < 0.001) and tibial components (*r* = 0.841; *P* < 0.001). Templating accuracy was high, with 92.7% of femoral and 88.7% of tibial components within ± 1 size. No significant differences in correlation strength or mean absolute deviation were observed across training levels (*P* > 0.05). The proportion of cases with > ± 1 size deviation was low across all groups and did not differ significantly between training levels for either component (femoral: *P* = 0.874; tibial: *P* = 0.791).

**Conclusion:**

2D digital templating for primary TKA demonstrated high accuracy, with reliable prediction within a ± 1 size range and no significant influence of surgical training level. These findings support its continued clinical use and confirm that templating can be reliably performed by residents at all stages of training.

Video Abstract

**Level of evidence:**

Level III, diagnostic study.

**Supplementary Information:**

The online version contains supplementary material available at 10.1186/s42836-025-00356-5.

## Introduction

Optimal outcomes in total knee arthroplasty (TKA) depend on multiple factors, including precise implant positioning and appropriate component sizing, both of which influence joint mechanics, soft-tissue balance, and long-term implant survivorship [1–3]. Therefore, rigorous preoperative planning is essential for achieving reliable clinical and functional results after TKA [4].

Preoperative planning typically involves radiographic templating to estimate appropriate component sizes [5]. Although advanced planning tools can improve precision [6, 7], two-dimensional (2D) digital templating remains the most widely used method in clinical practice due to its accessibility, efficiency, and low cost [8]. However, reported accuracy rates vary substantially across studies, and no universal standard exists regarding how templating should be performed or who should perform it [9].

In academic teaching hospitals, preoperative templating is often delegated to orthopaedic residents at various stages of training. While this involvement is essential for surgical education, it raises an important clinical question: Does the level of training influence the accuracy of templating, and is it acceptable to entrust this responsibility to residents without compromising planning quality? To date, little evidence exists on how experience level affects templating accuracy in TKA, and no guidelines define the level of supervision required.

The primary objective of this study was to assess the accuracy of 2D digital templating for primary TKA. The secondary objective was to evaluate whether the level of surgical training affects templating accuracy. We hypothesized that (1) planned and implanted component sizes would be strongly correlated, and (2) templating accuracy would differ significantly across training levels.

## Material and methods

### Patients

All patients who underwent primary TKA at a single academic orthopaedic center between October 2020 and March 2023 were retrospectively screened for eligibility. A total of 481 patients were eligible. Patients were eligible for inclusion if they had manual (non-robotic-assisted) implantation of the Attune knee system (Attune, DePuy Synthes) using either a cruciate-retaining (CR) or posterior-stabilized (PS) design, and if preoperative 2D digital templating had been performed using TraumaCad (Version 2.5; Brainlab AG, Munich, Germany). Additional inclusion criteria required the availability of complete electronic medical records and written informed consent.

**Fig. 1:**
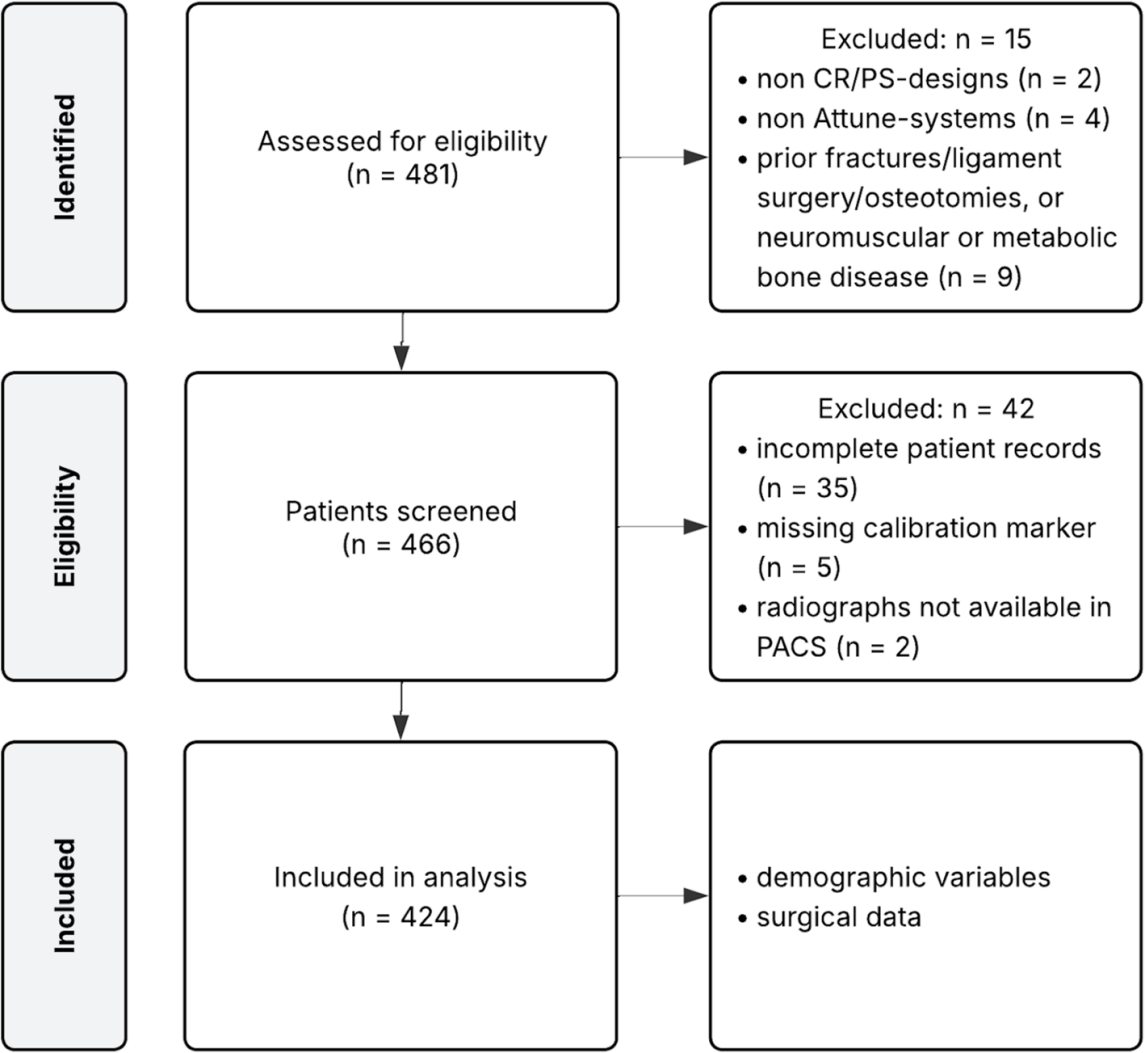
Flow chart of patient selection. *CR* = *cruciate-retaining; PACS* = *picture archiving and communication system; PS* = *posterior-stabilized*

Exclusion criteria included the use of any implant system other than Attune, absence of a calibration marker on preoperative radiographs, a history of fractures, previous ligament reconstruction or corrective osteotomies involving the affected knee, as well as underlying neuromuscular or metabolic bone disorders. Additionally, cases were excluded if templated radiographs were not uploaded to the institutional picture archiving and communication system (PACS) and thus unavailable for intraoperative digital access. The patient inclusion process is depicted in Fig. 1.

Demographic data (age at surgery, sex, side, and body mass index [BMI]) and surgical details (date of surgery, implant design, and femoral and tibial component sizes, surgeon’s information) were extracted from the patients’ electronic medical records.

### Preoperative templating

Preoperative planning was based on standardized standing anteroposterior (AP) and lateral digital radiographs of the operative limb. Imaging was acquired at the time of surgical indication using a digital radiography system (XGEO GC85A, Samsung, Seoul, South Korea) by our institution’s Department of Radiology and was calibrated with a 25-mm (1-inch) reference marker. For the AP view, calibrated radiographs were obtained in a standardized, true AP position with the patella centered between the femoral condyles. The knees were fully extended, feet positioned 10 cm apart and externally rotated by 10°, both according to a foot-positioning template, with hands placed alongside the body and equal weight distribution on each leg. To ensure full extension, the radiological technician instructed patients to engage their quadriceps and maintain an upright posture during imaging. Excessive knee rotation, defined as a non-centered patella or fibular head overlap exceeding one-third, was considered an exclusion criterion. Three sequential images were acquired and automatically stitched into one composite image using dedicated software (S-Station, Version 3.05; Samsung). For the lateral view, short-weight-bearing lateral-to-medial radiographs of the respective knee were obtained at the time of surgical indication. Radiographs were standardized by aiming for approximately 120° of knee flexion, with the detector positioned parallel to the sagittal plane and the X-ray beam centered on the patellofemoral joint line. No corrections were applied regarding the Coronal Plane Alignment of the Knee classification [10].

Digital 2D templating was performed in TraumaCad by orthopaedic surgeons or orthopaedic surgery residents, following the manufacturer’s instructions. Completed templates were uploaded to the institutional PACS (Centricity RIS-I 4.2 Plus; GE Healthcare, USA) and were digitally available for review in the operating room. The Attune system includes femoral and tibial components ranging from size 1 (smallest) to size 10 (largest), with optional narrow femoral components available for sizes 3 to 6.

### Surgical technique

All surgeries were performed by multiple surgeons using a standard medial parapatellar approach under tourniquet control. Implant fixation was achieved either through cementation or by the press-fit technique, and either a CR or PS design of the Attune TKA system was utilized. Surgical technique and component sizing were carried out in strict accordance with the manufacturer’s instructions. In cases where both preoperative templating and intraoperative evaluation indicated two viable component sizes, final selection was based on intraoperative assessment, including optimal bone coverage, appropriate rotational alignment, and balanced soft-tissue tension, to ensure an optimal fit.

### Statistics

Descriptive statistics are presented as means ± standard deviations and ranges for continuous variables, and as medians with interquartile ranges (IQR) for ordinal variables (implant sizes). Normality of data distribution was assessed using the Shapiro–Wilk test, and homogeneity of variances was evaluated with Levene’s test. For analysis, narrow femoral components (e.g., 5 N) were treated as their corresponding base size (e.g., 5). Implant accuracy was reported separately for femoral and tibial components and combined as “exact” when both planned and implanted sizes matched. Deviations were further categorized as ± 1, ± 2, and ± 3 sizes. Correlation analyses were performed between the planned and implanted femoral and tibial component sizes.

To evaluate whether templating accuracy differed by surgical training level, cases were stratified into three groups: junior residents (residents with < 3 years of orthopaedic residency training), senior residents (residents with ≥ 3 years of orthopaedic residency training), and attending surgeons (board-certified orthopaedic surgeons). The three-year threshold was chosen as it represents the midpoint of orthopaedic residency in Germany, which has a minimum duration of six years. Two analyses were performed: (1) Pearson correlation coefficients were calculated between planned and implanted component sizes for femoral and tibial components separately within each experience group, and (2) a one-way analysis of variance (ANOVA) was conducted to compare the mean absolute deviation between planned and implanted sizes across the three experience groups, separately for femoral and tibial components. Further, the proportion of cases with a deviation greater than ± 1 size between planned and implanted components was calculated separately for the femoral and tibial components across all three groups. Group differences were analyzed using the chi-square test of independence.

For all correlations, the correlation coefficient (*r*) and corresponding *P*-values were reported. Correlation strength was interpreted as follows: 0–0.19 = very weak, 0.20–0.39 = weak, 0.40–0.59 = moderate, 0.60–0.79 = strong, and 0.80–1.0 = very strong [11]. All analyses were two-tailed, and a *P* < 0.05 was considered statistically significant. Bonferroni correction was applied for multiple comparisons where appropriate.

Data were compiled and summarized using Microsoft Excel (version 16.78, Microsoft Corporation, Redmond, WA, USA), and all statistical analyses were conducted using IBM SPSS Statistics (version 28.0, IBM Corp., Armonk, NY, USA).

### Ethical aspects

This study was approved by the institutional ethics committee (approval number EA2/016/21) and conducted in accordance with the Declaration of Helsinki. Written informed consent was obtained from all participants prior to enrollment, and all patients received comprehensive information about the study’s purpose and potential risks.

## Results

A total of 424 patients (259 females [61%], 165 males [39%]). Demographic and clinical characteristics are summarized in Table 1.

**Table 1:** Patient demographics

Parameter	Mean ± SD or *n* (%)
Age, years	73.53 ± 9.06
BMI	31.05 ± 5.48
Sex	
Male	164 (39)
Female	260 (61)
Side	
Right	245 (58)
Left	179 (42)
Implant Type	
CR	345 (81)
PS	79 (19)

*SD* standard deviation, *BMI* body mass index, *CR* cruciate retaining, *PS* posterior stabilized

The median planned component sizes were 6 (IQR, 5–7; range, 3–10) for the femoral component and 6 (IQR, 5–7; range, 3–10) for the tibial component; the median implanted sizes were 6 (IQR, 5–7; range, 3–10) and 6 (IQR, 4–7; range, 3–10), respectively. Among all implanted femoral components, 43 (10.1%) were narrow designs.

Templating accuracy, reported as exact matches and deviation of ± 1, ± 2, and ± 3 sizes, is summarized in Table 2. Planned and implanted component sizes were very strongly correlated for both the femoral (*r* = 0.864; *P* < 0.001) and tibial component (*r* = 0.841; *P* < 0.001).

**Table 2:** Accuracy of preoperative 2D templating

Deviation category	Both components*, *n* (%)	Femoral component, *n* (%)	Tibial component, *n* (%)
Exact (0)	106 (25)	220 (52)	176 (43)
± 1 size	345 (81)	390 (92)	373 (88)
± 2 sizes	416 (98)	424 (100)	414 (98)
± 3 sizes	424 (100)	424 (100)	424 (100)

^*^“Both components” refers to a case-level measure, indicating that both the femoral and tibial component deviations fall within the specified category (e.g., “ ± 1 size” requires both components to be within ± 1 of the planned size). Deviation is defined as the absolute difference between planned and implanted size; narrow femoral components (e.g., 4 N) were classified according to their base size (e.g., 4 N → 4)

Of the included cases, 158 (37%) were planned by junior residents, 164 (39%) by senior residents, and 102 (24%) by attending surgeons. All experience groups demonstrated very strong correlations between templated and implanted sizes (*r* > 0.8 for both components; Table 3).

**Table 3:** Correlation analysis of the accuracy of preoperative 2D templating according to surgeon skill level

Surgeon skill level	*r*, (*P*-value) femoral component	*r*, (*P*-value) tibial component
Junior residents	0.861, (*P* < 0.001)	0.839, (*P* < 0.001)
Senior residents	0.872, (*P* < 0.001)	0.846, (*P* < 0.001)
Attending surgeons	0.866, (*P* < 0.001)	0.842, (*P* < 0.001)

Correlation analysis between planned and implanted size, with narrow sizes used as the base size, e.g., 4 N → 4. *r* = Pearson correlation coefficient

The mean absolute deviation between planned and implanted component sizes was comparable across all three groups. One-way ANOVA revealed no significant differences in templating accuracy between experience levels for either the femoral or tibial component (*P* > 0.05; Table 4).

**Table 4:** Deviation between planned and implanted size according to training level

Component	Mean absolute deviation ± SD	*P*-value (One-way ANOVA)
Femoral	Junior residents: 0.68 ± 0.62	*P* = 0.682
	Senior residents: 0.61 ± 0.66	
	Attending surgeons: 0.62 ± 0.63	
Tibial	Junior residents: 0.70 ± 0.65	*P* = 0.743
	Senior residents: 0.63 ± 0.67	
	Attending surgeons: 0.65 ± 0.66	

Correlation analysis between planned and implanted size, with narrow sizes used as the base size, e.g., 4 N → 4. *r* = Pearson correlation coefficient

The proportion of cases with a deviation greater than ± 1 between planned and implanted components was low across all levels of surgical training for both the femoral component (8.2% in junior resident cases, 7.3% in senior resident cases, and 6.9% of attending surgeon cases) and tibial (11.4%, 10.4%, and 9.8%, respectively) (Table 5). Chi-square analysis revealed no statistically significant difference between the groups for either component (femoral component: *P* = 0.874; tibial component: *P* = 0.791).

**Table 5:** Cases with > ± 1 size deviation by training level

Training level	Femoral > ± 1 (*n*, %)	Tibial > ± 1 (*n*, %)	Total Cases (*n*)
Junior residents	13 (8)	18 (11)	158
Senior residents	12 (7)	17 (10)	164
Attending surgeons	7 (7)	10 (10)	102
**Total**	32 (8)	45 (11)	424

Correlation analysis between planned and implanted size, with narrow sizes used as the base size, e.g., 4 N → 4. *r* = Pearson correlation coefficient

## Discussion

This study evaluated the accuracy of 2D digital templating in primary, manual TKA, and assessed whether orthopaedic surgery training level influences this accuracy. The key findings were that (1) preoperative templating and final implanted component sizes were very strongly correlated for both the femoral and tibial components, and (2) templating accuracy did not significantly differ between junior residents, senior residents, and board-certified attending surgeons. These results confirm our first hypothesis and reject the second, indicating that 2D templating is highly reliable and not dependent on the level of surgical training.

The accuracy of digital 2D templating for TKA remains inconsistently reported in the literature. While some studies describe relatively low accuracy with limited predictive value [9, 12–15], a recent systematic review by Lee et al. [8] highlighted high accuracy rates for both femoral and tibial components. Our findings support this, demonstrating very strong correlations between planned and implanted component sizes for both the femur (*r* = 0.864) and tibia (*r* = 0.841). Importantly, however, both our data and prior studies agree that 2D templating is not perfectly precise, with exact size prediction remaining limited. In our cohort, the exact match rate was 51% for the femoral and 42% for the tibial component, consistent with previous reports [8]. Nevertheless, the predictive accuracy within a corridor of ± 1 size was high, 92% for femoral and 88% for tibial components, supporting the clinical utility of 2D templating as a reliable tool for preoperative planning.

This level of accuracy is particularly relevant given the logistical, environmental, and economic advantages associated with effective size prediction [16, 17]. Even when exact matches are not achieved, a narrow-predicted range can help reduce implant inventory requirements, lower operating room turnover times and sterilization costs, benefits that are especially valuable in high-volume or resource-limited settings. While more advanced tools such as 3D templating, artificial intelligence, and deep learning algorithms have shown potential for improving accuracy [18–24], their broader implementation is currently limited by cost, infrastructure demands, and availability. A prospective cost-effectiveness analysis comparing these advanced technologies to standard 2D templating could provide valuable insight into whether the potential gains in precision justify the additional investment. For now, given its accessibility and reliability within a clinically useful margin of error, 2D digital templating remains an effective and sufficiently accurate method for preoperative planning in TKA [20].

Several studies have investigated potential factors influencing the accuracy of 2D digital templating in TKA. While BMI and surgical experience have not been associated with reduced accuracy, evidence suggests that sex may play a role, with higher accuracy reported in male patients [9, 25]. A study by Hsu et al. [26] evaluated templating accuracy across medical students, physician assistants, residents, and fellowship-trained arthroplasty surgeons and reported excellent intra-observer reliability across all examiners for 48 TKA patients. While our findings partly align with their results, the present study substantially extends the existing evidence by including a much larger cohort of 424 patients and examining templating performance in a real-world setting. Specifically, to our knowledge, we are the first to compare templating accuracy between junior residents, senior residents, and board-certified orthopaedic surgeons, groups that routinely perform or supervise preoperative planning in academic centers. No significant differences were found in either mean absolute deviation or correlation strength between planned and implanted sizes across training levels in our study. In addition, the proportion of cases with deviation greater than ± 1 size was low in all groups and did not differ significantly. These results suggest that even early-stage residents, when following standardized planning protocols, can achieve templating accuracy comparable to that of experienced surgeons.

This has important implications for both clinical efficiency and orthopaedic training. In teaching hospitals, it is common and often necessary to delegate tasks to residents. Our data support the safety and validity of this practice, showing that planning quality is not compromised by surgical training level. Delegating templating responsibilities can enhance workflow efficiency, promote resident autonomy, and allow senior staff to focus on other tasks without sacrificing planning accuracy.

## Limitations

This study has several limitations that should be acknowledged. First, it was conducted retrospectively at a single academic institution using one implant system (Attune, DePuy Synthes) and one digital planning platform (TraumaCad, Brainlab AG). As a result, the findings may not be fully generalizable to other implant systems, templating software, institutional workflows, or patient demographics. Second, only one implant system and a single digital planning platform were used, which may restrict the applicability to other systems. Third, final implant sizing was determined intraoperatively at the discretion of the operating surgeon, introducing a degree of subjectivity into the comparison. Furthermore, variables such as bone morphology, alignment deformities, or demographic factors were not included in the analysis. Furthermore, no 3D imaging or templating control group was included for comparison. While 3D-based planning methods may offer higher precision, their clinical use remains limited, and our findings therefore primarily reflect real-world 2D templating performance. Lastly, due to the design of this study, inter- and intra-observer reliability was not evaluated, so the reproducibility of templating results between observers remains unclear.

## Conclusion

2D digital templating for primary TKA demonstrated high accuracy, with reliable prediction within a ± 1 size range and no significant influence of surgical training level. These findings support its continued use in clinical practice and confirm that residents can perform templating reliably.

## Data Availability

The datasets generated and analyzed during the current study are available from the corresponding author upon reasonable request.

## Abbreviations

2D: Two-dimensional
ANOVA: Analysis of variance
AP: Anteroposterior
BMI: Body mass index
CR: Cruciate-retaining
IQR: Interquartile ranges
PACS: Picture archiving and communication system
PS: Posterior-stabilized
TKA: Total knee arthroplasty
